# Mr. White, on Opiate Friction

**Published:** 1801-11

**Authors:** W. White

**Affiliations:** Bath


### *To the Editors of the Medical and Physical Journal.*

GENTLEMEN,

IF you think the following case worthy a place in the Medical and Physical Journal, it is at your service. I am, &c.

*Bath, Oct.* 10, 1801. W. WHITE.

---

SEPT. 12. Eliz. Hall, ætat. 23, was seized five weeks ago with a chilliness, pain of her bowels and head. Three or four days after a nausea and vomiting came on, (of a yellow colour and bitter taste) accompanied with a diarrhœa. She was thirsty, skin hot, pulse quick, tongue white, had sweatings at night. Some calomel, and a saline cordial mixture, were prescribed for her; and, in the course of a fortnight, she appeared mending for three or four days. The sickness and vomiting however returned again, with stitches in different parts of the chest, and considerable oppression at the præcordia, greatly interrupting respiration. Notwithstanding every attention was paid to her, the complaint increased, and she was now so extremely reduced, that persons around her frequently thought she was expiring. Nothing had stayed on her stomach for several days, either of food or medicine. The vomiting was æruginous; she had singultus often; frequent loose motions, of a dark colour; pulse very feeble and quick; tongue florid, smooth, and shining; she was perfectly sensible.

In this deplorable situation, I directed her stomach and bowels to be fomented with a decoction of camomile flowers, and afterwards a liniment formed of ℥ss tinct. opii and a yolk of an egg, one half of which was rubbed on the abdomen* at two o'clock, p. m. and the remainder at bed-time. What is very remarkable, she never was sick, nor vomited after the first friction,

---

* The liniment could not be applied immediately to the region of the stomach, as she had a blister on three or four days before, which was not quite healed.

tion, but fell aſleep immediately on the application of the opium, and ſlept for ſome time. The fomentation and friction were continued a few days, gradually decreaſing the quantity of opium; and as her ſtomach could bear it, a little wine was frequently given. It may be obſerved, that not only the vomiting ceaſed, but the diarrhœa alſo; and ſhe had no complaint afterwards, except a ſlight pain of her legs. Her pulſe was reduced to 96 on the third day, which before was exceedingly quick.

She is recovering her ſtrength very faſt.

